# Tennessee Veterinary Medical Association

**Published:** 1897-03

**Authors:** 


Secretary.

### TENNESSEE VETERINARY MEDICAL ASSOCIATION.

A meeting of the graduates of veterinary medicine and surgery in Tennessee was held at Nashville, February 9th, at the office of Drs. Rayen and Plaskett, for the purpose of organizing an Association. Quite a number of local veterinarians as well as visiting members were present. Dr. W. C. Rayen was appointed temporary chairman. The following officers were elected: President, Dr. J. W. Scheibler, Memphis; First Vice-President, Dr. Joseph M. Good, Chattanooga; Second Vice-President, Dr. H. D. Fenimore, Knoxville; Secretary, Dr. Joseph Plaskett, Nashville; Treasurer, Dr. T. W. Scott, Nashville. The afternoon meeting was occupied in an interesting discussion of rare and unusual cases that had come under the observation of the members present, and some time was devoted also to the consideration of new drugs.

The next meeting will be held at Nashville, just prior to the meeting of the United States Veterinary Medical Association.

### NORTH CAROLINA VETERINARY MEDICAL ASSOCIATION.

